# A Bayesian multi‐stage modelling framework to evaluate impacts of energy development on wildlife populations: an application to greater sage‐grouse (*Centrocercus urophasianus*)

**DOI:** 10.1016/j.mex.2023.102023

**Published:** 2023-01-18

**Authors:** Brian G. Prochazka, Shawn T. O'Neil, Peter S. Coates

**Affiliations:** U.S. Geological Survey, Western Ecological Research Center, Dixon Field Station, 800 Business Park Drive, Suite D, Dixon, CA 95620 USA

**Keywords:** Anthropogenic development, Bayesian, Before-after-control-impact, Decision support tool, Geothermal energy, Population matrix model, Sagebrush ecosystem, Sage-grouse, Survival analysis, State-space model, *Multi-stage modelling to evaluate impacts of energy development on wildlife populations with applications to landscape conservation planning*

## Abstract

Increased demand for domestic production of renewable energy has led to expansion of energy infrastructure across western North America. Much of the western U.S. comprises remote landscapes that are home to a variety of vegetation communities and wildlife species, including the imperiled sagebrush ecosystem and indicator species such as greater sage-grouse (*Centrocercus urophasianus*). Geothermal sources in particular have potential for continued development across the western U.S. but impacts to greater sage-grouse and other species are unknown. To address this information gap, we describe a novel two-pronged methodology that analyzes impacts of geothermal energy production on pattern and process of greater sage-grouse populations using (a) before-after control-impact (BACI) measures of population growth and lek absence rates and (b) concurrent-to-operation evaluations of demographic rates. Growth and absence rate analyses utilized 14 years of lek survey data collected prior (2005–2011) and concurrent (2012–2018) to geothermal operations at two sites in Nevada, USA. Demographic analyses utilized relocation data, restricted inference to concurrent years, and incorporated 17 additional control sites. Demographic results were applied to >100 potential geothermal sites distributed across the study region to generate spatially explicit predictions of unrealized population-level impacts.

• State-space and generalized linear models yield estimates of population growth and lek absence rates, respectively, before and after the onset of geothermal energy production; distances ranging from 2–30 km are evaluated as alternative control-impact footprint hypotheses; this provides inference about the spatial extent as well as the magnitude of impacts associated with geothermal development.
• Estimation of important population demographic rates are implemented to investigate the processes by which geothermal energy development might reduce population growth; independent estimates of confounding, environmental effects from 17 control sites are made spatially explicit within ‘impact’ models to establish baseline conditions otherwise masked by collinearity.
• Population matrix models are built using estimates from demographic analyses to provide landscape mapping of impacts associated with potential geothermal sites.

## Graphical abstract

**Figure:**
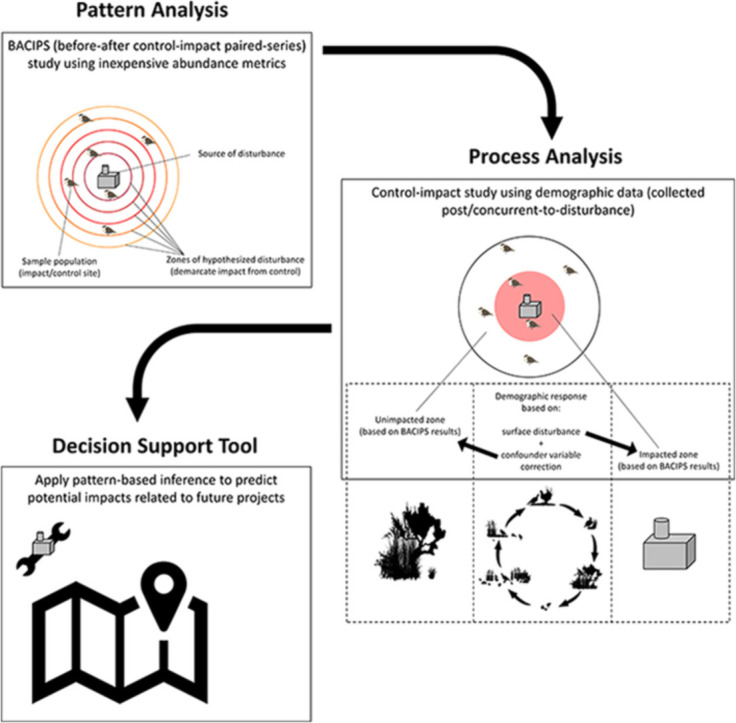

Specifications table

**Table:** 

Subject Area:	Agricultural and Biological Sciences
More specific subject area:	*Energy development and ecosystems*
Method name:	*Multi-stage modelling to evaluate impacts of energy development on wildlife populations with applications to landscape conservation planning*
Name and reference of original method:	*N.A.*
Resource availability:	R 3.4.3. R Core Team (2020). R: A language and environment for statistical computing. R Foundation for Statistical Computing, Vienna, Austria. https://www.R-project.org/.
	Plummer, Martyn (2018). Rjags: Bayesian graphical models using MCMC. R package version 4-8. https://CRAN.R-project.org/package=rjags.
	Prochazka, B.G., S.T. O'Neil, P.S. Coates, and M.P. Chenaille (2022). Median estimates of impact potential from geothermal energy production activities on greater sage-grouse populations, Nevada and California (2022): U.S. Geological Survey data release, https://doi.org/10.5066/P9OLC725.

## Investigating population patterns – a before‐after control‐impact study design (Analysis 1)

The initial stages of establishing a relationship between energy development projects and wildlife populations ideally involves some version of a before-after control-impact (BACI) study design. Here, we used a BACI framework to assess changes in population patterns (growth rates and extirpation [absence] probabilities) as a function of distance to surface disturbances associated with energy development. Population indices were collected from multiple, fixed locations representing sub-populations (hereafter, population nodes) and spanned multiple breeding seasons. Importantly, population nodes were spaced in a manner that allowed the testing of distance thresholds in relation to the surface disturbances. Population indices were assigned to treatment (i.e., impact vs control) and period (i.e., before vs after onset of surface disturbance) categories based on spatiotemporal attributes. Period attributions were fixed for all analyses, whereas treatment attributions varied based on multiple competing hypotheses, which made different assumptions about the size of the impacted area around the surface disturbance. Population nodes that fell ‘outside’ the hypothesized zone of impact were modeled as a control group, whereas population nodes that fell ‘inside’ the zone were modeled as the impact group. Specifically, we calculated the Euclidean distance between population nodes and the nearest point or edge of any infrastructure associated with the surface disturbance and then assigned population nodes to control or impact groups based on a particular distance threshold. Multiple thresholds were tested, resulting in a set of “candidate” zones.

### Population growth rates

The population indices used for this example are counts of individuals from multiple locations (population nodes). However, the list of alternative indices is essentially inexhaustible, limited only by the availability of data and the user's statistical and technical expertise. Counts were assumed to occur over a series of years, before and after the onset of energy development. Furthermore, population nodes were assumed to exist over a relatively large area surrounding energy development infrastructure with substantial variation in position respective of that infrastructure. This monitoring scenario allows for a quantification of surface disturbances at multiple spatiotemporal scales, providing greater insight into potential conflicts with wildlife populations. The first method contrasted trends in population abundance between candidate impact and control zones, before and after the onset of energy development activity. Trends were evaluated using predicted relative abundance (N) and intrinsic rate of change (r) from Bayesian hierarchical state-space models (SSM; [1,2]), which partition variance components into latent state processes and observation errors [1]. Detection of individuals during wildlife surveys is often considered imperfect (i.e., detection probability is < 1.0; [3]). However, SSMs can provide unbiased estimates of r and an index of N when observation error is constant across years [1,4]. Log-transformed count data (y) for unique population nodes (l) and years (j) was assumed to follow a Normal distribution:

(1) $$log\left(y_{lj}\right)\;∼\;\text{Normal}\left(log\left(N_{lj}\right),\;\sigma_{obs_{l}}^{2}\right)$$

Parameters N and r were estimated for l and j using the expression:

(2) $$log\left(N_{l,j+1}\right)=log\left(N_{lj}\right)+r_{lj}$$

We assumed $r_{lj}$ was distributed normally about a mean ($\mu_{r_{lj}})$ with population node-specific interannual variation ($\sigma_{r_{l}}^{2}$):

(3) $$r_{lj}\;∼\;\text{Normal}\left(\mu_{r_{lj}},\sigma_{r_{l}}^{2}\right)$$

Eqs. (2) and (3) represent the state process while Eq. (1) links the true state to the observations (log-transformed annual maximum counts). We assigned vague priors to the initial size of each population node. We fit $\mu_{r_{lj}}$ as a deterministic function of constant and varying effects [5], expressed as:

(4) $$\mu_{r_{lj}}=\;\alpha_{l}+\beta_{tp}+\eta_{j}$$

which included varying effects for each population node ($\alpha_{l}$) and year ($\eta_{j}$), as well as constant effects for each period-treatment combination ($\beta_{tp}$). Varying effects were centered on zero and assigned diffuse, group-level variance priors to account for individual and temporal variation, respectively. Subscripts for the constant effect $\beta_{tp}$ reference the assignment of population node (l) and year (j) to a particular treatment (t) and period (p) category. The combination of treatment and period categories were modeled as four distinct groups (i.e., before-control, before-impact, after-control, after-impact). Support for each candidate zone was tested using approximate leave-one-out cross-validation methods for Bayesian models with Pareto smoothed importance sampling (PSIS-LOO CV) [6]. Analyses were run in program R, (version 3.4.0; R Core Team, 2017) using the ‘loo’ package [6]. The lowest LOO information criterion (LOOIC) value indicated which candidate impact zone possessed the greatest support.

We evaluated evidence of energy development impacts using BACI ratios [7] and CI measures [8] applied to posterior distributions of λ (i.e., $e^{r}$) across the set of candidate impact zones. BACI ratios provide test statistics that quantify effect sizes of positive or negative impact [7]. To calculate BACI ratios, we first used λ to derive ratios of impact to control (i:c) before ($R_{\lambda ,\;i:c,\;b})$ and after ($R_{\lambda ,\;i:c,\;a})$ the onset of energy development.

We derived estimates of impact by calculating the ratio between time periods, expressed as:

(5) $$R_{\lambda ,\;BACI}=R_{\lambda ,\;i:c,\;b}/R_{\lambda ,\;i:c,\;a}$$

These test statistic ratios have advantages over other BACI techniques because they directly contrast probabilities of change in an area of impact relative to controls [7]. Additionally, incorporating full posteriors provides accurate estimates of uncertainty for the ratios. We determined weak, moderate, and substantial evidence of energy development impacts if 85, 90, and 95% of the highest probability density exceeded a ratio of 1.0, respectively.

We also calculated two control-impact (CI) measures, termed CI-contribution and CI-divergence [8], which indicate whether impact zones or control zones experienced change between time periods (CI-contribution) and the degree of dissimilarity between impact and control zones (CI-divergence). The CI-contribution is expressed as:

(6) $$\text{CI}-\text{contribution}\;=\left|\lambda_{i,a}-\lambda_{i,b}\left|-\right|\lambda_{c,a}-\lambda_{c,b}\right|$$

Positive values for CI-contribution reflect changes in impact zones evidencing effects of energy development, whereas negative values reflect changes in control zones that might otherwise confound interpretation of BACI ratios. The CI-divergence is expressed as:

(7) $$\text{CI}-\text{divergence}=\left|\lambda_{i,a}-\lambda_{c,a}\left|-\right|\lambda_{i,b}-\lambda_{c,b}\right|$$

Positive values for CI-divergence reflect greater dissimilarity between impact and control zones following energy development, whereas negative values reflect greater similarity between impact and control zones following energy development. BACI ratios combined with CI measures provide a rigorous investigation of energy development impacts and were estimated across all candidate impact zones for comparison and evaluation of distance effects.

### Population absence rates

In a second BACI analysis, we investigated changes in probability of population absence following multiple years of energy development activities. Here, count data collected at each population node were subset to exclude all but the final two years of before and after periods. For example, a scenario consisting of 30 years of before (1–15) and after (16–30) data would result in the retention of years 14–15 (before period) and 29–30 (after period). This particular subset seeks to answer two questions: (1) was the population active immediately before the onset of energy development activities (year 16) and (2) was the population active 14–15 years after the onset of energy development activities. We defined absence as the occurrence of zero counts for both years within the same period. Survey of the second year of each before and after period served as a confirmation of absence. Thus, if the second year's count provided evidence of activity, the population node was scored as -“presence.” We modeled absence (A) before and after the onset of energy development activities within control and impact zones for each specified candidate zone to investigate evidence of population node inactivity resulting from the energy development project. Increases in the probability of population node absence, relative to controls, indicates changes in the distribution and/or number of individuals on the landscape stemming from energy development projects.

We carried out the same BACI ratio test statistics applied to λ to estimate the effect of energy development on A. To estimate A for each before-after and control-impact category across each candidate impact zone, we used generalized linear models with a Bernoulli distribution, $y_{l\;}∼\;Bernoulli\left(p_{l}\right)$, and logit link function,$\;logit(p_{l})$, where y was measured as a binary response for each population node (l) and was either absent (1) or present (0) within each of the four possible BACI groups (impact-before, impact-after, control-before, control-after). We modeled each distance-treatment demarcation separately and used PSIS-LOO CV to identify the best supported distance demarcation for absence effects.

All models of λ and A were specified in JAGS (just another Gibbs sampler; version 4.2.0) using package *rjags* which implements MCMC algorithms [9]. We used three independent chains of 50,0000 sample iterations and discarded the initial 40,0000 iterations as burn-in. We thinned chains by a factor of 10, thereby retaining 3000 samples to comprise each posterior distribution. We examined evidence of Markov chain convergence using the R-hat statistic (≤ 1.1; [10]) and history plots.

## Investigating population processes – a concurrent‐energy development control‐impact study design (Analysis 2)

If monitoring of individual demographic performance occurs during energy development operations (within and outside the zone of impact), a subsequent step can be implemented to determine which processes are contributing to changes in the distribution and/or size of the impacted population. The primary reason for monitoring outside the zone of impact (i.e., using a control group) is to establish a cause-and-effect relationship between the dependent (e.g., survival) and independent (e.g., distance to energy development) variables of interest. Additional independent variables will undoubtedly have cause-and-effect relationships with the dependent variable. However, the control group corrects for nuisance sources of variation by being similar to the impact group in all ways except for the independent variable of interest. To effectively isolate the independent variable of interest, monitoring protocols must be standardized across control and impact groups such that additional sources of difference do not infiltrate the experimental design. The same rationale applies to environmental conditions and the choice of control groups must take these into consideration as well.

During this step we developed a population matrix model within a Bayesian framework that identified energy development impacts on specific vital rates using a two-stage (control-impact) modeling process. The first stage of the two-stage process involved modeling background sources of variation attributed to environmental conditions (referred to as ‘confounder covariates’) for each demographic rate using data collected from study sites that were not associated with energy development projects. This step was done to estimate environmental effects (i.e., parameters) in the absence of energy development, and ultimately formed a baseline prediction (at every MCMC iteration) for each animal location within energy development sites, given local environmental conditions (i.e., locally recorded data). During stage 2, a second likelihood, run concurrently (concurrent to confounder likelihood) and within the same JAGS wrapper function, was used to estimate energy development-related covariates in the presence of confounder predictions (i.e., the product of control-informed parameter(s) and locally recorded data). We performed these two stages for each vital rate model, thereby alleviating issues of spatial autocorrelation among confounder and geothermal covariates. Furthermore, the method accounts for the full uncertainty in confounder effects by estimating a new baseline likelihood at every iteration of the model.

### Covariate reduction and model building

For each demographic subcomponent model, we used a sequential-stage approach to build a generalizable model [2,11], to deal with potentially large numbers of plausible confounder covariates and correlations among spatial extents and distance effects. We first carried out the covariate reduction approach for the confounder variables in stage one by choosing the spatial extent with the highest prediction accuracy among each spatial extent for each land cover type using PSIS-LOO CV. We also retained the most supported energy development effects using PSIS-LOO CV.

Of those variables carried forward, we developed a full model with all the top confounder covariates and the main and interaction terms of the top geothermal covariates. For all demographic subcomponent models, non-informative priors were placed on the confounder covariates. In the case of energy development covariates (including interaction terms) we assigned conditional priors using the Bayesian Lasso [12] and indicator variable selection [13] techniques. The Bayesian Lasso used a Laplace (i.e., double exponential) distribution with diffuse hyperprior, which was uniformly distributed between 0–10. The indicator variable selection technique was specified using a Bernoulli distribution with prior inclusion probability (*p*) set to 0.75 for main effects (i.e., lower order terms). In the case of higher order terms (i.e., interactions), we employed the strong heredity principle [14], which assigns an additional conditional prior probability of inclusion dependent upon the presence of all lower order terms. Evidence for important coefficients are presented as Bayes Factors [15] with strength of evidence evaluated according to Kass and Raftery [16].

For Bayesian Lasso implementation, we ran models on four chains of 100,000 iterations and removed the initial 50,000 iterations as burn-in. For PSIS-LOO CV model runs we obtained 3000 posterior MCMC sample iterations from three chains of 50,000 iterations thinned by a factor of 10 following a burn-in of 40,000 iterations. All models were implemented in JAGS through program R. Chain convergence was assessed using history plots and R-hat statistics.

### Demographic models

Demographic models chosen for Analysis 2 can vary based on the focal species, data availability, and user preference/knowledge. For illustrative purposes we assume a complex life history of an avian species.

#### Model for annual survival

We modeled annual survival ($j_{an}$) as a continuous process observed at discrete intervals (e.g., daily, weekly, monthly). Individual status was determined as dead or alive during each relocation event. Individuals were right censored under 3 circumstances: (1) the study period ended before death was determined; (2) the method for relocation was no longer possible (e.g., transmitter failure); or (3) the animal could no longer be relocated because it moved outside of the study area. We considered censoring a random process and all individuals either died or were right censored. Graduation from one age class to the next was made possible through the specification of a time-varying covariate.

During the first stage, we used uniquely-marked individuals from independent sites with no energy development activity to model survival using Bayesian shared frailty models [17], [18], [19] and estimated coefficients (β) for multiple confounder covariates (C) given data (X) on survival, which took the form:

(8) $$UH_{C,\;aijk}=\;exp\left(\sum_{c=1}^{C}\beta_{C}X_{C,aijk}+\Psi_{a}+\;Z_{k}+\;\Gamma_{i}+\;\Lambda_{j}\right)$$

We refer to Eq. (8) as the ‘control’ likelihood for animal survival and identify it as such using the subscript C. The unit hazard (*UH*) was modeled as a deterministic function of constant and varying effects [5]. We chose to estimate effects as constant when we believed the effect to be identical for all groups in a population and varying otherwise. Site and year were treated as varying effects, and their variances as parameters that were estimated from the data. Constant effects included confounder and energy development covariates as well as age and month. A baseline log hazard was estimated for each interval ($Z_{k}$) to account for seasonal variation in the survival process. We also estimated separate coefficients for each age class ($\Psi_{a}$) to account for potential differences at that level. Subpopulation ($\Gamma_{i}$) and year ($\Lambda_{j}$) were included to account for spatiotemporal patterns, and those took the forms, $\Gamma_{i}\;∼\;Normal\left(0,\;\sigma_{\Gamma }^{2}\right)$ and $\Lambda_{j}∼\;Normal\left(0,\;\sigma_{\Lambda }^{2}\right)$. Subscripts a*,* i*,* j*,* and k reference age, subpopulation, year, and month, respectively. The control likelihood was used to account for confounder effects that were unrelated to energy development activity, but still influence and explain environmental stochasticity in population dynamics by contributing to local habitat structure and composition.

At each iteration of the Markov chain we derived a predicted log unit hazard ($LUH_{P}$) for each animal location at the energy development sites using confounder effects ($B_{C}$, Ψ, *Z*, Λ) estimated in the control likelihood and their complementary covariates measured at the energy development sites ($X_{C^{{}^{▪}}},a^{▪},k^{▪},j^{▪}$), which was expressed as:

(9) $${LUH}_{P,aijk}=\sum_{c=1}^{C}B_{C}X_{C^{▪},aijk}+\Psi_{a{}^{▪}}+Z_{k^{▪}}+\Lambda_{j^{▪}}$$

The predicted unit log hazard (9) represented sources of variation in survival present at, but not confounded by energy development operations, thereby preventing effects of energy-related covariates from influencing estimation of background environmental factors. In a parallel process (at every MCMC iteration) we estimated the unit hazard at energy development sites ($UH_{G}$) as a deterministic function of the predicted log unit hazard ($LUH_{P}$), constant effect for energy development site (Ω), and energy-based covariates (*G*). The mean effect size of control sites was set to zero so that it could be excluded from predictions. That equation took the form:

(10) $$UH_{G,aijk}=\;\text{exp}\left(LUH_{P,aijk}+\sum_{g=1}^{G}\Delta_{G}X_{G,aijk}+\;\Omega_{i}\right)$$

The cumulative hazard (*CH*) and survival function (*S*) were subsequently expressed as:

(11) $$CH_{\;aijk}=\;\sum_{k=1}^{T}UH_{1:k,aij}$$

and:

(12) $$S_{\;aijk\;}=e^{-CH_{aijk}}$$

#### Model for second nest propensity

We modeled the propensity to initiate a second nest ($\lambda_{np2}$) as a Bernoulli process where the random variable (y) took on a value of 1 when a hen initiated a nest following the first attempt and 0 if it did not. The discrete probability distribution for second nest attempts at energy development sites was expressed as:

(13) $$y_{G,ij\;}∼\;Bernoulli\left(p_{G,\;ij}\right)$$

where subscripts i and j represent subpopulation and year, respectively. The logit-transformation, $logit(p_{G,hij})$, was modeled using a similar two-stage approach as previously described. Baseline predictions (*P*) were informed by confounder effects estimated at multiple control sites, and energy-related covariates were explored using the equation:

(14) $$logit\left(p_{G,\;ij}\right)=\;P_{ij}+\sum_{g=1}^{G}\Delta_{G}X_{G,ij}+\Omega_{i}$$

Again, Ω represents a constant effect for each energy development site (i), subscript j references individual year, $\Delta_{G}$ is a vector of energy development effects, and $X_{G}$ a matrix of energy development covariates.

#### Model for egg hatchability

We modeled egg hatchability ($\lambda_{h}$) using a binomial distribution where the number of successes was represented by the number of hatched eggs (y) and the number of trials as the initial clutch size (n). Observations were recorded at the clutch (e) level for every subpopulation (i) and year (j). At the energy development sites that equation was expressed as:

(15) $$y_{G,\;eij\;}∼\;Binomial\left(p_{G,\;eij},\;n_{G,\;eij}\right)$$

The logit-transformation representing probability of success, $logit(p_{G,\;eij})$, was modeled using a similar two-stage approach as described previously. Baseline predictions (P) informed by local conditions and confounder effects estimated from control sites were combined with impact site ($\Omega_{i}$) and energy development effects ($\Delta_{G}$) using the equation:

(16) $$logit\left(p_{G,\;eij}\right)=\;P_{eij}+\sum_{g=1}^{G}\Delta_{G}X_{G,eij}+\Omega_{i}$$

#### Model for clutch size

It is possible that certain life stages will be unaffected by energy development activities. Here, we assumed that energy development would not have an impact on clutch size. Under these circumstances, it is not necessary to estimate confounder covariates at control sites. However, it is necessary to estimate or specify (e.g., informative prior) the parameter for population matrix modeling steps (see below). Therefore, first and second nest clutch size estimates ($N_{\text{cs}1}$ and $N_{\text{cs}2}$) were derived in this example using a single likelihood. Observation of clutch size at energy development sites ($y_{G_{\text{cs}}}$) were assumed to arise from a Poisson distribution as follows:

(17) $$y_{G_{cs}}∼\;Poisson\left(N_{cs,at}\right)$$

where the log expected count of clutch size was a deterministic linear function of effects for age (a), and nest attempt (t). That equation took the form:

(18) $$\log\left(N_{cs,at}\right)=\Psi_{a}+T_{t}$$

#### Model for nest survival

We derived first ($\varphi_{\text{ns}1}$) and second ($\varphi_{\text{ns}2}$) nest survival parameters using a shared frailty model, which was similar in structure to Eqs. (8)–(12). We employed the same two-stage modeling process and sequential modeling approach as described for other demographic rates to evaluate energy development effects while accounting for environmental sources of variation in nest survival at control sites. Within this model we fit hen as a varying effect to account for individuals that were monitored at more than one nest. We also estimated an effect of nest attempt to account for differences in survival owing to that level of variation. Encounter histories were constructed using daily intervals and we subsequently derived a cumulative hazard that spanned the full duration of the egg-laying and incubation periods.

#### Model for chick survival

We estimated chick survival ($\varphi_{cs}$) probabilities using chick counts conducted at 10-day intervals up to 50 days post hatch. The same modeling approach described for egg hatchability Eqs. (15)–((16)) was applied to the chick survival dataset with two key exceptions: (1) the random variable (y) now represented the number of chicks observed at 50 days post hatch and (2) the number of trials (n) represented the initial brood size (i.e., number of eggs hatched).

#### Models for unobserved life stages

Certain life stages may preclude observation for a multitude of reasons. To demonstrate the ability of the framework to accommodate such circumstances, we specified two life stages (initial nest propensity, $\lambda_{np1_{a}}$; juvenile survival, $\varphi_{js}$) using informative priors and based them off literature values from comparable populations [20].

#### Population matrix model

We linked all demographic models as subcomponents in an age-structured population matrix model [21] and derived relative total population abundance ($N_{T}$) for each year at energy development sites using estimates of annual survival (*φ*) and fecundity (g), which was expressed as:

(19) $$N_{T_{i,j+1}}=\frac{\Sigma_{a=1}^{A}N_{T_{ija}}g_{ija}}{2}+\Sigma_{a=1}^{A}N_{T_{ija}}j_{an,ija}$$

We derived g for each age (a), subpopulation (i), and year (j) using the equation:

(20) $$g_{ija}=\left(\lambda_{np1_{a}}\lambda_{c1_{ija}}\varphi_{ns1_{ija}}\lambda_{h_{ija}}\varphi_{cs_{ija}}\varphi_{js}\right)+\;\left(\left(1-\varphi_{ns1_{ija}}\right)\lambda_{np2_{a}}\lambda_{c2_{ija}}\varphi_{ns2_{ija}}\lambda_{h_{ija}}\varphi_{cs_{ija}}\varphi_{js}\right)$$

and used a divisor of 2 (Eq. (19)) because we assumed equal sex ratios at hatch [22]. We then derived finite rate of population change ($\lambda_{P}$) as:

(21) $$\lambda_{P_{i,j+1}}=\frac{N_{T_{i,j+1}}}{N_{T_{ij}}}$$

The population matrix model allowed us to investigate relative impacts and potential for cumulative impacts across each demographic rate and $\lambda_{P}$.

## Example using real data

### Project description and study area

The methods described in this study were used to evaluate impacts of geothermal energy development on greater sage-grouse (*Centrocercus urophasianus;* hereafter sage-grouse), an important avian indicator species of sagebrush ecosystem health in western North America. A two-pronged analysis was employed, with the first analysis focused on modeling (a) differences in population growth rates and lek absence rates before and after the onset of geothermal activities, and (b) the most explanatory distance at which these impacts were detected. The second analysis was focused on evaluating concurrent-to-geothermal operation influences on population demographic rates of sage-grouse, such as survival (adult, juvenile, nest, and chick), and other components of reproduction (nest propensity, hatchability, and clutch size). Following analyses of demographic rates, a population matrix model was used to predict impacts of geothermal energy infrastructure at sites where infrastructure currently does not exist but could in the future based on high degrees of geothermal energy potential.

**Fig. 1:**
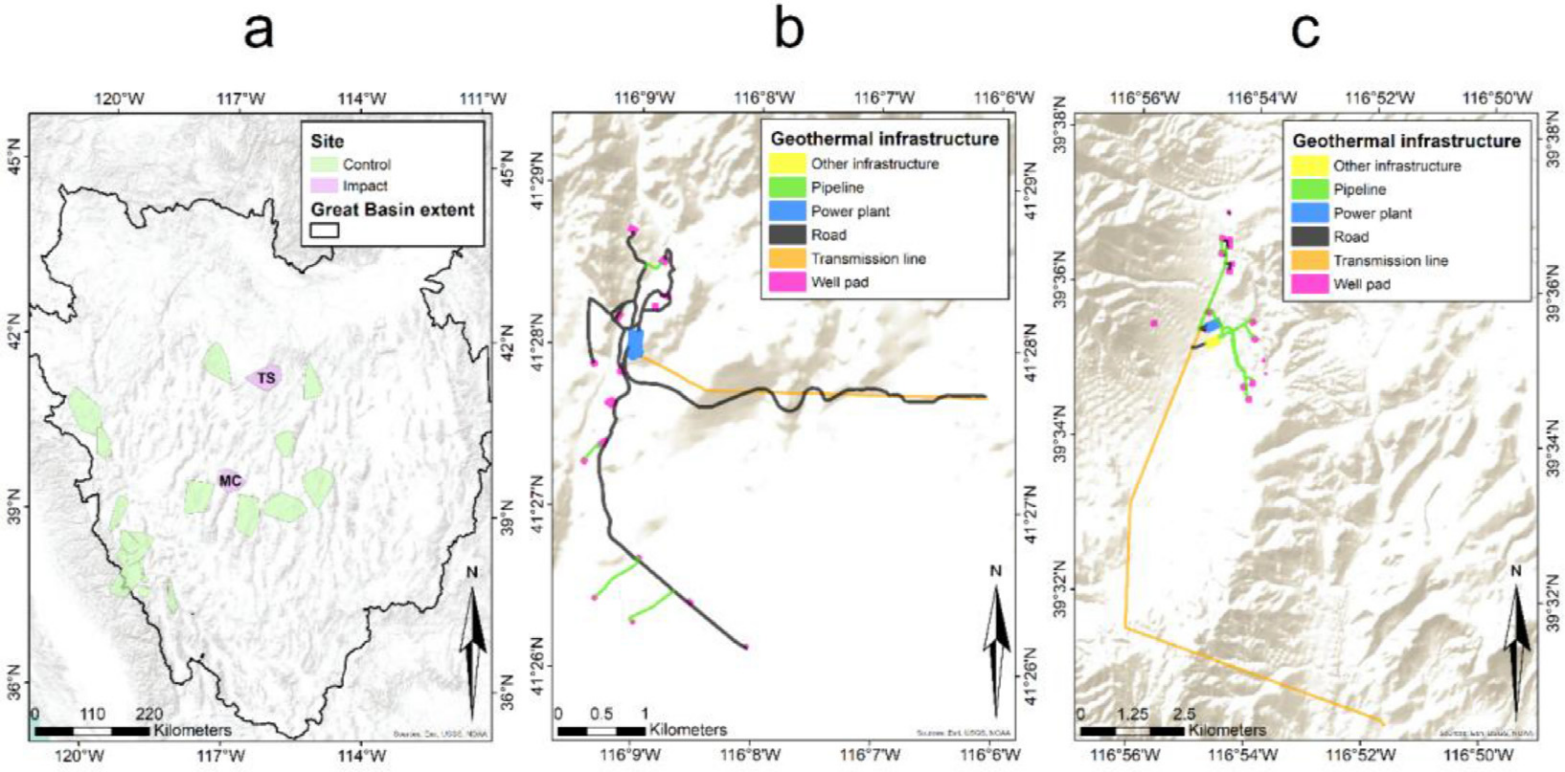
(a) Seventeen control sites and two impact (geothermal) sites located within the hydrographic Great Basin (solid black line). Geothermal infrastructure associated with the (b) Tuscarora Mountains (TS) and (c) McGinness Hills (MC) geothermal power plant facilities. Infrastructure identified by colored polygons was constructed prior or concurrent to the monitoring of local populations used to model demographic parameters in this study (2012–2018).

Sage-grouse were monitored at two primary study locations, Tuscarora Mountains and McGinness Hills, where recent geothermal energy development occurred (Fig. 1a). The Tuscarora geothermal site (TS) was located in northern Elko County in Independence Valley, approximately 115 km north of Elko, Nevada (Fig. 1b). The southern study area was the McGinness Hills site (MC) located in Lander County approximately 15 km northeast of Austin, Nevada (Fig. 1c). Both sites contained numerous forms of above ground infrastructure including drilled wells (production and injection) and pipelines (above and below ground), which transferred water to and from the power plants [23]. Pipelines generally paralleled existing roads, but some new two-track access roads were developed along pipeline infrastructure. Each site was comprised of turbines, condensers, and cooling towers capable of producing 18 (TS) and 143 (MC) MW net capacities [24]. Direct surface disturbance from geothermal production (e.g., plants, wells) at TS and MC covered ∼25 and ∼88 ha, respectively. Power transmission lines and access roads were developed for each facility within these interspaces. The TS site included a 40 km, 120 kV transmission line with ∼44 ha of surface disturbance on private and public lands (Bureau of Land Management; hereafter, BLM), while a 14.5 km, 230 kV transmission line with access roads on private and public lands occurred at the MC site, resulting in ∼27.8 ha of surface disturbance [23]. Exploration activities and construction of geothermal infrastructure started in 2008, and associated power plants, transmission lines, roads, and production wells were completed by 2011. Additional infrastructure was added to both facilities between 2012–2018; these additions were considerably smaller than the original surface disturbances.

Sage-grouse were monitored from 2012–2018 at 17 independent field sites representing distinct subpopulations within sagebrush ecosystems of Nevada and California (Fig. 1a). These study sites were not impacted by geothermal development and served to establish baseline predictions of environmental factors that influence sage-grouse population vital rates (e.g., nesting, chick survival, and adult survival) and were generalizable to the geothermal sites where the same factors were likely confounded by varying degrees of collinearity with geothermal infrastructure. Comparisons of confounder covariate distributions among control and impact sites revealed strong similarities with fewer than 0.05% of values recorded at impact sites falling outside the range of control counterparts. The widespread distribution of control sites allowed for appropriate representation of estimated relationships between environmental factors and sage-grouse population vital rates. The northern and western sites received relatively more precipitation than other sites and typified sagebrush steppe [25]. The southwestern sites were at relatively higher elevations on the boundary of California and Nevada. The central sites were typical of sagebrush semi-desert [25] with relatively drier and warmer soil types. Generally, each study site was dominated by big sagebrush (*Artemisia tridentata*) and little sagebrush (*A. arbuscula*), with a heterogeneous mixture of saltbrush species at low elevations which included greasewood (*Sarcobatus baileyi*), horsebrush (*Tetradymia spp.*), and rabbitbrush (*Chrysothamnus and Ericameria spp.*). High elevations consisted of mountain shrub-steppe communities that included big and little sagebrush as well as ephedra (*Ephedra spp.*), serviceberry (*Amelanchier spp.*), snowberry (*Symphoricarpos spp.*), and antelope bitterbrush (*Purshia tridentata*). Non-woody plants included grasses such as basin wild rye (*Leymus cinereus*) and Idaho fescue (*Festuca idahoensis*), and forbs such as balsamroot (*Balsamorhiza spp.*) and lupine (*Lupinus spp.*). Single-leaf pinyon (*Pinus monophylla*) and Utah juniper (*Juniperus spp.*) stands were interspersed with varying densities, as were patches of invasive cheatgrass (*Bromus tectorum*). All areas were topographically diverse with elevations ranging from 1,158–3,770 m.

### Application of Analysis 1 to sage‐grouse lek count data

We carried out a before-after control-impact paired (BACI) study design using lek count data collected at both geothermal sites from 2005–2018. Leks are traditional breeding grounds where sage-grouse congregate over an 8–12-week period (March–May). Interannual fidelity to leks provides opportunities to count male sage-grouse, which serve as a widely recognized abundance index [26,27]. Each year, we sought to conduct 3–4, 10 min surveys per lek site throughout the lekking period so that the collection of annual surveys for each lek overlapped peak attendance [28]. Lek counts were conducted between 30 min before and 90 min after sunrise using binoculars or spotting scopes at locations with views of the entire lek.

Counts were recorded from all known leks (*n*=103) located within 60 km of geothermal infrastructure. We used a distance-based sampling approach so that control leks represented the same ecoregion with similar functional habitat types as that of impact zones. We assessed six different distance thresholds (i.e., candidate impact zones) to identify the impact zone that garnered greatest support from the data. Three candidate distances were based on studies relating energy development to sage-grouse populations, including ∼2 km [29,30], 5 km [31,32], and 10 km [33]. We included three additional candidate impact zones (15, 20, and 30 km) to evaluate sequentially larger areas and avoid possible truncation of a distance effect. The maximum distance for any lek in the analysis (57.2 km) allowed an adequate sample of leks for the control group regardless of the selected impact zone (control group = leks at distances greater than selected impact zone up to 57.2 km). Number of leks assigned to the control group for each of the six candidate zones were 96 (>2 km), 92 (>5 km), 89 (>10 km), 82 (>15 km), 78 (>20 km), and 53 (>30 km).

At both geothermal sites, we completed 14 years of lek surveys following published methodologies and protocols [34] in collaboration with state and federal agencies. This yielded sample intervals of 7 years before (2005–2011) and 7 years after (2012–2018) the onset of geothermal operations. All lek count data underwent quality assurance and quality control (QA/QC) procedures and maximum annual counts were retained and used in analyses.

Lek count data used to evaluate population growth rates and lek absence rates were modeled according to the methods described for Analysis 1 (sections 1.1. & 1.2.). SSM assumptions of interannual constancy for observation errors was verified prior to modeling [3]. Lek count data used to estimate lek absence rates were subset to exclude all but the final two years of before (2011–2012) and after (2017–2018) periods. We chose 2011–2012 to represent the ‘before’ period because counts from those years occurred immediately before (2011) or coincident to (2012) energy development activities. Years 2010–2011 would have been more appropriate for representing the before period. However, samples near the plant (i.e., within 5 km) were limited given the consecutive year ruleset. Had the 2010–2011 years been used to assess lek absence, more than 80% of leks within 5 km would have been removed owing to lack of data (i.e., counts were not conducted in 2010). Conversely, fewer than 20% of leks within 5 km needed to be removed based on a 2011–2012 assessment of before-period lek activity. Moving further back in time (e.g., 2009–2010) presented similar issues as 2010–2011 and going too far back would have been inappropriate given the irrelevance of historic lek activity information on contemporary assessments. While this choice of before period could impose some bias in the estimation of lek absence, we found no evidence for it when evaluating the 36 leks that had count data collected during every year of the 2010–2012 period. Using that subset of leks, 100% were considered active under the 2010–2011 and 2011–2012 assessments.

### Application of analysis 2 to sage‐grouse demographic data

From 2012–2018, we conducted extensive on-the-ground monitoring of individual female sage-grouse (*n*=1,049) using telemetry techniques at the two geothermal sites (*n*=230) and the 17 other study sites (*n*=819) without geothermal development to investigate survival and reproduction. We captured sage-grouse using standard spotlighting techniques [35] during spring and late summer/fall and outfitted them with necklace-style VHF radio-transmitters (Advanced Telemetry Systems, Isanti, MN) equipped with mortality signals that pulsed on after ≥8 h of sage-grouse inactivity. Sage-grouse age was determined as yearling (>1 and <2 years) or adult (≥2 years) by examining wing feather characteristics [36].

We sought to relocate sage-grouse twice per week during the reproductive season (March–September) and once per month during fall and winter (fixed-wing aircraft). In the field, sage-grouse were circled from 30–50 m. We visually confirmed nests from females found in the same position on two consecutive relocations [37]. Nest confirmation was conducted with binoculars at ∼10 m distance from nests to avoid disturbing the female. Clutch size at active nests was recorded opportunistically during visits that coincided with nest recesses or visits that resulted in the incidental flushing of a female. These values were later used for clutch size and hatchability estimates. Nests were monitored at least twice weekly until nest fate could be determined. We considered nests successful if ≥1 egg hatched, whereas failures were evidenced by depredation or abandonment. Following failed nests, we continued monitoring sage-grouse at least twice weekly to confirm renest locations using the same procedures as for initial nests. Following completion of successful nests and renests, brood-rearing females were relocated, and chicks were counted [38,39] every 10 days until the brood reached 50 days post-hatch. We sought to avoid flushing sage-grouse during chick surveys. If no chicks were detected, an additional survey was conducted within 24–48 h to confirm failure. For each demographic model, we excluded data when (1) sage-grouse were not relocated following capture and transmitter deployment; (2) relocation dates were missing; (3) information regarding the status of nest, chicks, adult/yearling sage-grouse (i.e., dead or alive) were missing; or (4) unique identification of sage-grouse could not be determined. In addition, to evaluate predator densities, we conducted raptor-raven point count surveys from mid-March to mid-September from 2012–2018. Surveys were conducted within minimum convex polygons (MCPs) defined by annual sage-grouse radio telemetry locations.

Remotely sensed land cover covariates were evaluated to capture accurate estimation of geothermal operation effects by accounting for other environmental effects on demographic rates of sage-grouse. Specifically, we evaluated continuous percent cover of herbaceous perennial vegetation, little and big sagebrush, total shrub canopy (e.g., included non-sagebrush cover such as rabbitbrush and bitterbrush), annual grass, as well as sagebrush height using percent cover at the 30 m^2^ land cover data from National Land Cover Database [40]. We investigated tree canopy cover based on recent high-resolution mapping [41]. Percent cover of riparian and wet meadow areas were derived from LANDFIRE products [42], which are Landsat-based mapping products that consisted of binary classification at the 30 m^2^ resolution. We evaluated different extents of each covariate across each life stage by calculating percent cover within radii of 167.9 m (8.7 ha), 439.5 m (61.5 ha), or 1451.7 m (661.4 ha) using the neighborhood analysis tool (ArcMap 10.5; ESRI, 2018). These extents were derived from averages of the minimum, mean, and maximum daily movement distances of sage-grouse in the Great Basin [11]. For the nesting period we included 30-m and 75-m extents to reflect most immediate habitat based on movement patterns of nesting sage-grouse [43]. Evaluation of different spatial extents was appropriate because ecological processes influencing sage-grouse can be modulated based on scale of inference [44], [45], [46], [47]. We also investigated distance effects to water sources using National Hydrography Dataset [48]. Specifically, we calculated Euclidean and exponential decay distances to perennial and intermittent streams and springs following previously published procedures [11]. Lastly, we included topographic roughness, which was a measure of variability of the terrain [49], as it is known to influence sage-grouse habitat selection within the Great Basin [11].

At the geothermal sites, we evaluated additional covariates that may influence demographic rates. Site-level covariates included Euclidean distances from each sage-grouse nest, brood, and adult/yearling location to the nearest edge of the geothermal plant itself (hereafter ‘DGP’) and nearest edge of the geothermal footprint (e.g., road, powerline, pipeline, well, etc.; hereafter ‘DGF’). We compared DGF and DGP effects to determine whether the footprint (all development/infrastructure) or plant (primary source of light and noise) explained more variation in the data for each vital rate. To simplify model description, we generalize DGF and DGP as distance to geothermal infrastructure (hereafter ‘DGI’). However, DGF and DGP are referred to when referencing the top performing geothermal distance covariate for each vital rate. All infrastructure was digitized using high resolution aerial imagery (‘World Imagery’ in ArcMap 10.5; ESRI, 2018).

**Fig. 2:**
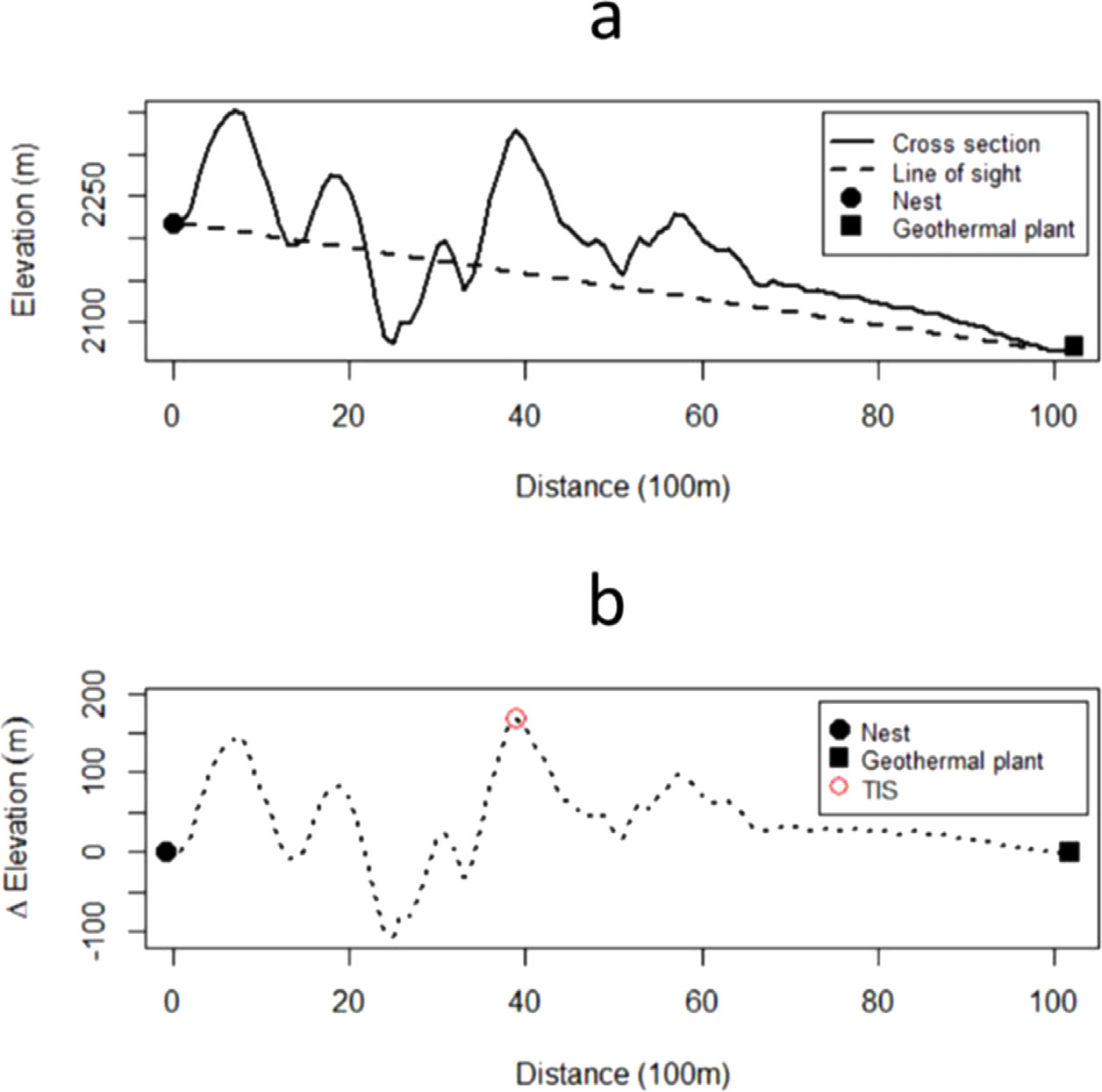
(a) Cross-section of a topographically rough surface and line of sight spanning a point of interest (e.g., nest location) and point source of light and sound waves (e.g., geothermal power plant). (b) The difference between line of sight and topographic cross section is used to demonstrate the calculation of a topographic impedance surface (TIS). The TIS value identified by a red circle (panel b) is assigned to the nest location.

Because geothermal power plants emit noise and light, we also carried out multiple steps to develop a continuous covariate that served as an index for these effects and refer to it as a topographic impedance surface (TIS). First, we created a minimum convex polygon (MCP) based on all telemetry locations across all years of study. We then created Euclidean spatial lines that connected every 30 m^2^ grid cell within the MCP (start cells) to the 30 m^2^ grid cell at the center of the geothermal power plant (end cell). Iterating through all spatial lines, one at a time, we extracted the elevation values for every cell intersected by the spatial line using a Digital Elevation Model (DEM; 30-m^2^ resolution). This provided us with varying elevations from each pixel point on the landscape to geothermal plant. We then subtracted those values from a baseline elevation that was derived between the start and end points (using a constant slope), which adjusted for the elevations of the two start and end points. Lastly, for each start cell we assigned the maximum difference along each Euclidean line. This procedure provided a continuous surface of topographic impedance (TIS) that was assumed to approximate dispersion of sound and light across the landscape from the geothermal infrastructure (Fig. 2). TIS and DGI were extracted to every telemetry point (nest, brood, general) for modeling the effects of geothermal infrastructure and activity on demographic rates.

Raptor-raven point count survey data were used to develop a raven density index (RDI) at each nest site location at the geothermal sites, largely because ravens are known to prey on sage-grouse eggs [37,50,51]. We were interested in more complex relationships between ravens and geothermal energy covariates given the species propensity for attraction to anthropogenic subsidies [51,52]. Therefore, at each of the geothermal sites, we conducted raven point count surveys from mid-March to mid-September from 2012–2018. Surveys were conducted throughout the breeding season at sage-grouse locations as well as randomly generated locations. Using distance sampling methods and distance-decay functions established from previously published research within the same broad study region [51], we developed the RDI to characterize spatial and temporal variation in relative raven densities associated with each sage-grouse nest. Surveys were conducted within MCPs defined by annual sage-grouse radio telemetry locations. We sought to perform ≥200 surveys for each field site each year and designed the timing and spatial distribution of surveys to coincide with sage-grouse breeding, nesting, and brood-rearing life phases. Specifically, surveys were conducted at locations used by sage-grouse (e.g., radio telemetry locations) as well as at randomly placed locations within each field site's survey boundary using a stratified random design to cover different land cover types as well as variation in distances to geothermal infrastructure. Survey methods followed those published elsewhere [53,54]. At each survey point, we recorded all observed ravens using binoculars and unaided eyes from a visual scan of the 360° viewscape. We recorded time, distance, and bearing of each raven using a watch, digital rangefinder, handheld GPS device, and compass, respectively.

The raven index was developed using hierarchical distance sampling methods [55,56]. Specifically, we employed a detection function that accounts for increasing detection failure as distance increases from the observer to the raven, which allows estimation of density, given an established search area. The search area was identified as the approximate distance at which detection probability declined below 0.1 (1.125 km; [57]). We estimated the detection function, g[r,z], for point data, where the probability of detecting ≥1 raven is conditional on distance (r) from the center of a survey point as well as the vector of possible covariates (z) that could influence detection [58]. We used a half normal key function [59,60] with area of viewshed and percent of forested landscape fit as covariates on the scale parameter of the detection function [58]. The viewshed estimated the visible proportion of the landscape based on the topography surrounding the observer and was evaluated using a DEM with 30-m spatial resolution. We also incorporated site and year group effects when supported by the available data (≥ 60 raven observations required for estimation of site, year, or year within site effect [57]), to account for variation in detection probabilities associated with differences in observers [51]. Raven observations were assigned to 5 distance classes, with cut-points at 225, 450, 675, 900 and 1,125 m [56]. We obtained parameter estimates for the distance-detection function using ‘unmarked’ [60] in R 4.0 [61]. Finally, we used the Horvitz-Thompson-like estimator [59] along with the parameters from our fitted detection function to calculate raven density (raven density index = RDI) for each individual nest location using the raven count data occurring within 3.5 km of the nest sites each year [59,62,63]. This procedure captured spatial and temporal variation in raven abundance within the extent of each monitored site, such that estimates for each sage-grouse nest were local (radius of 3.5 km based on approximate raven territory size; [52,64]) and time-specific, while also being quantified independently of potential confounding anthropogenic effects.

For each demographic subcomponent model, we used the sequential-stage approach described for Analysis 2 (sections 2.1.–2.10.). For our nest survival model, we incorporated RDI as an additional ‘geothermal’ covariate and investigated a three-way interaction between DGI, TIS, and RDI, which included all possible two-way interactions.

### Geothermal application tool

**Fig. 3:**
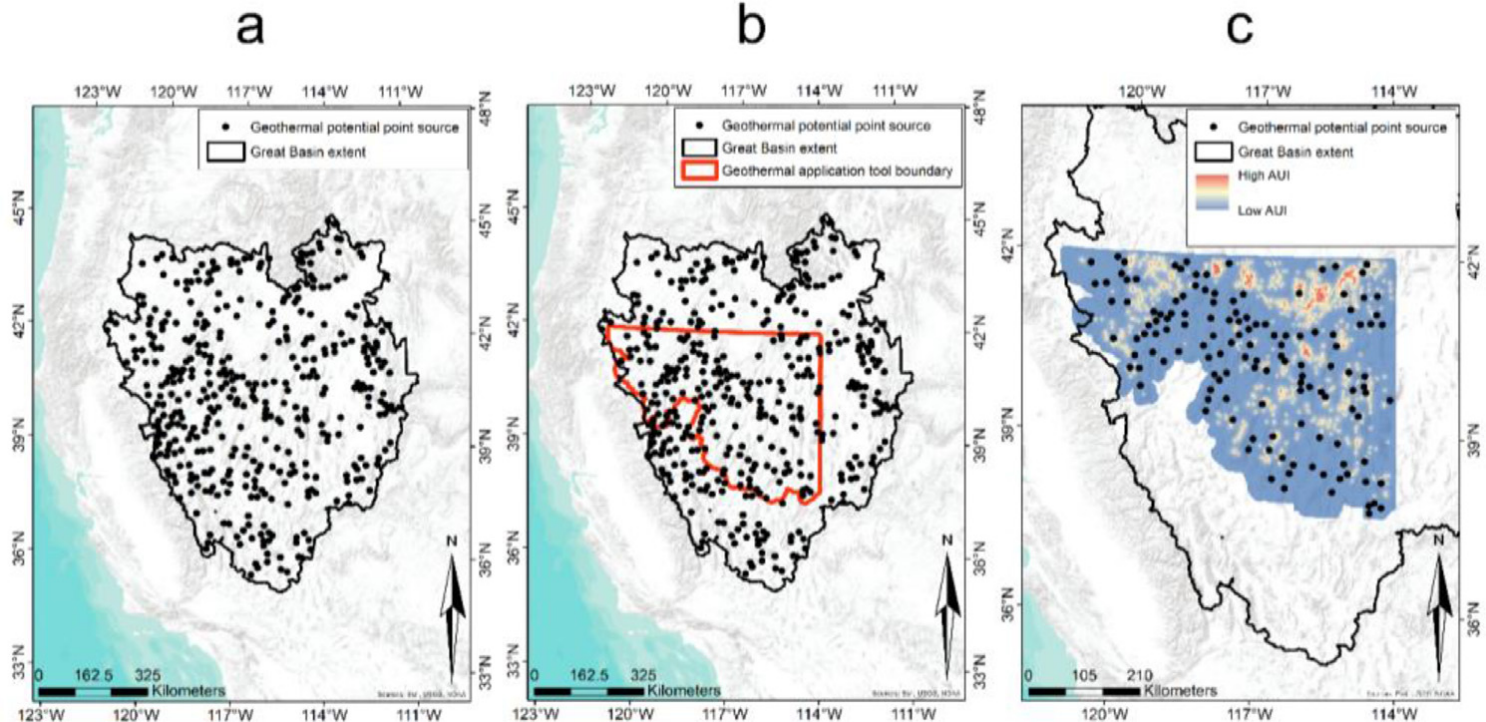
(a) Geothermal energy potential point sources that lie within the hydrographic Great Basin extent, (b) subset to include only those points that fall within the range of greater sage-grouse (*Centrocercus urophasianus*) in Nevada and northeast California, and (c) the same points overlaid on a greater sage-grouse abundance and space use index (AUI) layer.

In a final step, we demonstrate a quantitative approach using model predictions from Analysis 2 to help inform future planning of geothermal energy development as it relates to the conservation and management of sage-grouse populations. We first obtained areas of potential and existing geothermal activity throughout the Great Basin. We obtained geothermal data spanning the Great Basin from the Nevada Bureau of Mines and Geology [65] as point sources that were “active” or “under construction,” and from Mullane et al. [66] as point sources with MWh estimates for “beneficial heat”. We subset point sources to areas located within Nevada and northeast California. Due to clustering of many points within small areas, and the assumption that a single geothermal plant could utilize each source from a central location, we combined clusters of geothermal potential point sources that were within a 4-km radius of one another. We refer to these geothermal potential point sources (*n* = 135) hereafter as ‘candidate sites’ (Fig. 3c). We buffered candidate sites by 10 km, as this was beyond the maximum distance of effects from geothermal operations identified by Analyses 1 and 2. Spatially explicit surfaces (30 m resolution) were generated to support prediction for each environmental and geothermal variable that received model support from Analysis 2. Importantly, DGI and TIS were calculated for all candidate geothermal locations. We then obtained spatially explicit model predictions for demographic rates to derive *φ* (survival), *f* (fecundity), $\;N_{T}$ (total population abundance), and $\lambda_{P}$ (finite rate of population change) estimates across each buffered candidate site using the population matrix model developed in Analysis 2. Baseline prediction surfaces without geothermal effects were used to approximate ‘before’ conditions and predicted surfaces including geothermal effects approximated ‘after’ conditions. We subtracted the ‘after’ surface from the ‘before’ surface to produce a $\Delta \lambda_{P}$ surface. We then multiplied the $\Delta \lambda_{P}$ surface by a sage-grouse abundance and space use index surface (*AUI*) derived by Coates et al. [11]. The product of $\Delta \lambda_{P}$ and *AUI* [67] was summarized within 2, 5, and 10-km buffers and ranked in terms of greatest potential impact.

## Declaration of Competing Interest

The authors declare that they have no known competing financial interests or personal relationships that could have appeared to influence the work reported in this paper.

## Data Availability

Full Data Available: https://doi.org/10.5066/P9OLC725.
